# White adipose tissue undergoes browning during preweaning period in association with microbiota formation in mice

**DOI:** 10.1016/j.isci.2023.107239

**Published:** 2023-06-28

**Authors:** Anju Tsukada, Yuko Okamatsu-Ogura, Emi Futagawa, Yuki Habu, Natsumi Takahashi, Mira Kato-Suzuki, Yuko Kato, Satoshi Ishizuka, Kei Sonoyama, Kazuhiro Kimura

**Affiliations:** 1 Laboratory of Biochemistry, Faculty of Veterinary Medicine, Hokkaido University, Sapporo 060-0818, Japan; 2 Laboratory of Nutritional Biochemistry, Research Faculty of Agriculture, Hokkaido University, Sapporo 060-0809, Japan; 3 Laboratory of Food Biochemistry, Research Faculty of Agriculture, Hokkaido University, Sapporo 060-0809, Japan

**Keywords:** Endocrine system physiology, Microbiome

## Abstract

Beige adipocytes are transiently induced during early postnatal period in mice. Previous studies have suggested that, unlike in adults, the induction is independent of the sympathetic nerve activity; however, the mechanism is yet unknown. Here, we showed that beige adipocytes are induced during the preweaning period in association with the formation of microbiota in mice. Alteration of gut microbiota composition in preweaning mice by maternal treatment with antibiotics or high-fat diet feeding substantially suppressed WAT browning. The suppression was also found in pups transplanted cecal microbiota from pups of high-fat diet-fed dams. These treatments reduced the hepatic expression of genes involved in bile acid synthesis and the serum bile acids level. The abundance of Porphyromonadaceae and Ruminococcaceae in microbiota showed a positive and negative correlation with the induction of beige adipocytes, respectively. This finding may provide comprehensive understanding of the association between gut microbiota and adipose tissue development in the neonatal period.

## Highlights

• Beige adipocytes appear in WAT of neonatal mice but disappear after weaning
• Depletion or alteration of gut microbiota suppresses preweaning WAT browning
• Preweaning WAT browning is linked to changes in bile acid metabolism
• *Ucp1* expression correlates with Porphyromonadaceae and Ruminococcaceae

## Graphical abstract

**Figure:**
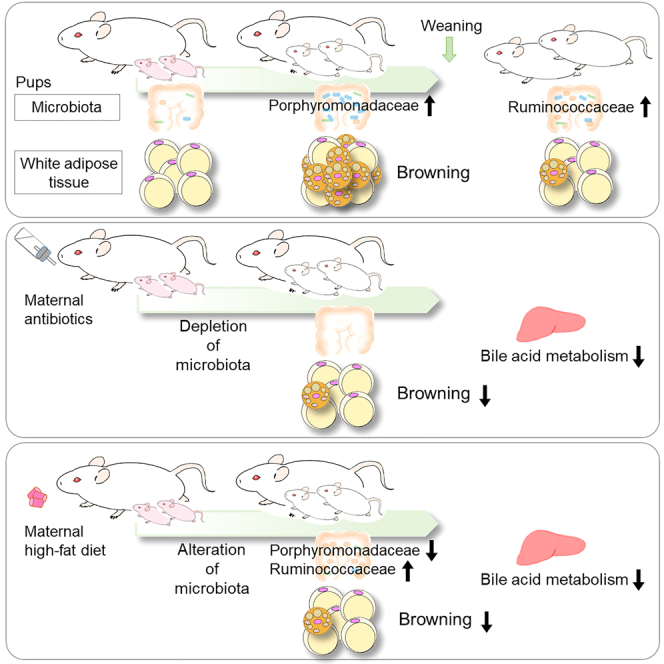

## Introduction

Adipose tissue in mammals is mainly classified into two types: white adipose tissue (WAT) and brown adipose tissue (BAT). WAT is located subcutaneously and intraperitoneally, and it consists of white adipocytes that store triglycerides as a large unilocular lipid droplet.^1^ BAT is primarily found in the interscapular and perirenal region in rodents, and it consists of adipocytes with multilocular lipid droplets and abundant mitochondria containing thermogenic uncoupling protein 1 (UCP1), which consumes energy as heat.^2^^,^^3^ The role of two adipose tissues in whole-body metabolism is contrasting. WAT stores excess energy as lipid, and enlargement of WAT caused by obesity induces systemic insulin resistance, leading to related metabolic diseases such as diabetes, hyperlipidemia, and cardiovascular diseases. By contrast, the activation of BAT increases whole-body energy expenditure dependently on UCP1 activity and prevents obesity in rodents and humans.^4^

The activation of UCP1 and its gene expression in BAT is induced by sympathetic stimulation such as cold exposure.^5^ When the cold exposure is prolonged, the expression of *Ucp1* is increased in BAT and induced in specific depots of WAT.^6^ Adipocytes expressing UCP1 in WAT are known as beige adipocytes, and this induction is referred to as browning of WAT.^7^ Beige adipocytes have considerable amount of UCP1 and thermogenic function comparable to that of brown adipocytes,^8^ and they regulate whole-body metabolism through secretion of factors such as cytokines, metabolites, and exosomes.^9^^,^^10^^,^^11^ Furthermore, beige adipocytes have attracted attention as a therapeutic target for obesity and related metabolic diseases because human BAT is mainly composed of beige adipocytes rather than brown adipocytes.^12^^,^^13^ Considering that human BAT declines with aging,^14^^,^^15^ finding ways to increase beige adipocytes is necessary.

WAT browning can be induced by stimuli such as cold exposure and overeating depending on the sympathetic nervous system activity in adult mice and rats as previously mentioned.^16^^,^^17^^,^^18^ In addition, beige adipocytes were transiently induced in WAT during the early postnatal period in mice.^19^ In the case of postnatal WAT browning, the sympathetic nervous system seems to be dispensable because newborn pups born and raised at cold (17°C) or warm (29°C) temperatures showed no difference in UCP1 induction in WAT.^20^ Moreover, beige adipocytes are induced before sympathetic development,^21^ and mice lack the network of sympathetic neurites preserved in the UCP1 expression in WAT,^22^ indicating that browning occurs independent of sympathetic nerves during the neonatal period. Thus, the mechanism of postnatal WAT browning in the preweaning period might be different from that in adult mice; however, this hypothesis remains unclear.

The neonatal period is important for the formation of gut microbiota. The intestinal tract is sterile in fetus, but it starts colonization immediately after birth when exposed by maternal or environmental factors.^23^ The gut microbiota is shaped and stabilized under the influence of breast milk, but the composition is easily affected by several factors such as diet, environment, and antibiotic treatment.^24^^,^^25^ The inhibition of forming healthy gut microbiota or the decrease in a specific type of bacteria leads to various diseases such as digestive disorders, autoimmune diseases, and obesity.^26^^,^^27^ The relation of microbiota to WAT browning has also been reported. Cold exposure changes microbiota composition in adult mice, and microbiota transplantation from cold-exposed mice to germ-free mice induces beige adipocytes.^28^ Another study has suggested that a shift in the gut microbiota is related to WAT browning induced by intermittent fasting.^29^ Thus, postnatal gut microbiota formation may play a role in WAT browning during the early postnatal period in mice.

In this study, we investigated the mechanism underlying the induction of beige adipocytes in the early postnatal period in relation to the formation of gut microbiota in mice.

## Results

### Postnatal change in white adipose tissue in mice

**Figure 1:**
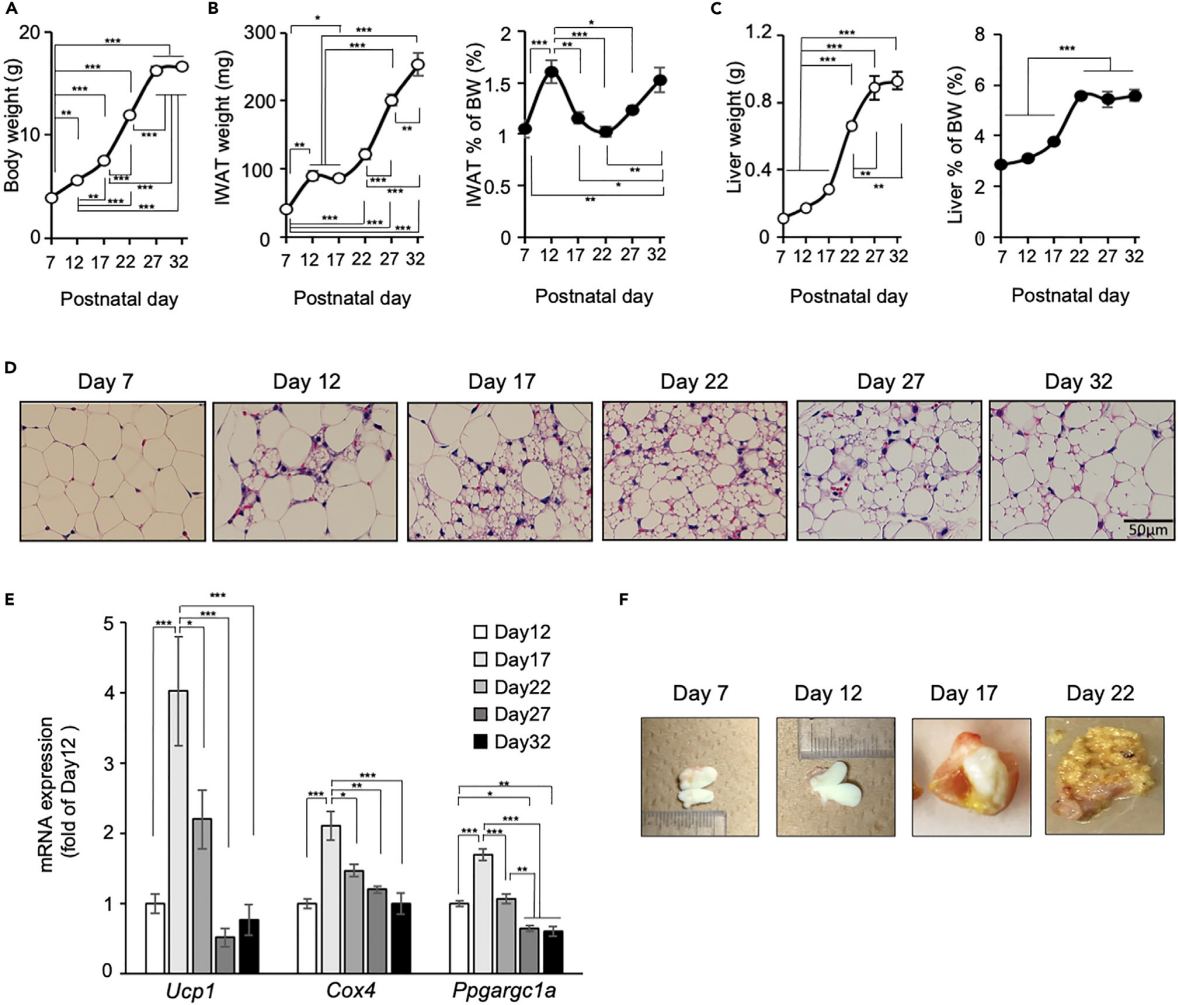
Postnatal change in white adipose tissue in mice (A–C) Postnatal changes in the body weight (A), tissue weight for inguinal white adipose tissue (IWAT) (B), and liver (C), and their ratio to body weightis shown. (D) Histological images of IWAT. Scale bar: 50 μm. (E) Gene expression of *Ucp1*, *Cox4*, and *Ppargc1a* in IWAT. The expression values were normalized to *Actb* and expressed relative to the value on day 12. (F) Gross images of stomach cut into half to observe the contents. All values represent means ± SEM (n = 4–5 per group, ∗p < 0.05, ∗∗p < 0.01, ∗∗∗p < 0.001, one-way ANOVA followed by Tukey’s Honest Significant Difference test).

In examining the postnatal development of WAT in mice, tissues were collected every 5 days from postnatal day 7 to day 32. The weights of inguinal white adipose tissue (IWAT) gradually increased age dependently as well as the body weight (BW), but the ratio to BW showed a transient increase on day 12 (Figures 1A and 1B) and a gradual increase after day 22. By contrast, the liver ratio significantly increased on day 22 and kept constant thereafter (Figure 1C). Histologically, IWAT consisted of adipocytes with a large unilocular lipid droplet, typical to white adipocyte, on day 7 (Figure 1D). On day 12, adipocytes containing multilocular lipid droplets were observed and surrounded by white adipocytes. The number of multilocular adipocytes increased until day 22 and then gradually disappeared thereafter. On day 32, a small number of multilocular adipocytes were observed, but their lipid droplets were larger in size compared with those before day 27. The expression level of brown/beige adipocyte marker *Ucp1* showed a transient and significant increase on day 17 and decreased thereafter (Figure 1E). The time-course of Ucp1 expression showed some differences to that of the appearance of multilocular adipocytes, which is possibly because of the time required for translation and the differences in half-life of Ucp1 mRNA and protein.^30^^,^^31^ The expression of *Cox4*, a mitochondrial marker, and *Ppargc1a*, a transcriptional cofactor important for transcription of *Ucp1*, showed a similar pattern depending on the postnatal day. These results indicate that beige adipocytes were transiently induced in IWAT during the postnatal period around day 17. In examining the timing of weaning, we compared stomach contents of pups. The stomach was filled with curd until day 12, and a small number of consumed pellets were observed on day 17. On day 22, only pellets were observed in the stomach content, indicating that pups were completely weaned (Figure 1F).

### Postnatal change in microbiota and its relation to *Ucp1* expression in IWAT

**Figure 2:**
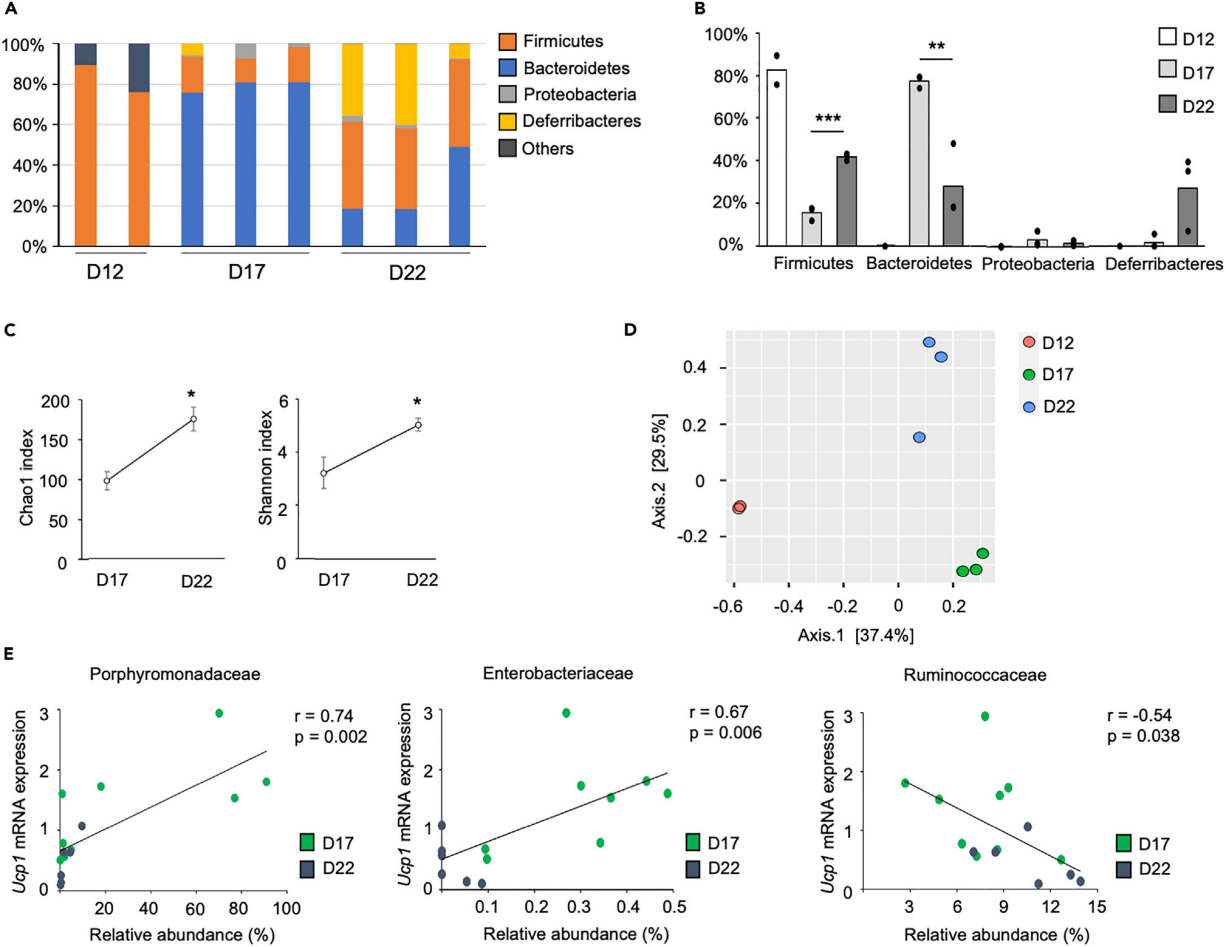
Postnatal change in microbiota and its relation to *Ucp1* expression in IWAT (A) The relative taxonomic abundance of bacterial phyla in the caecum of 12-, 17-, and 22-day-old pups. (B) Differences in the relative taxonomic abundance of major bacterial phyla. Considering that the low number of samples was succeeded in the amplification of 16S rRNA on day 12, only the data of days 17 and 22 were used for statistical analysis (n = 3 per group, ∗∗p < 0.01, ∗∗∗p < 0.001, Student’s *t* test). (C) Analysis of α diversity of Chao1 and Shannon indexes. Values represent means ± SEM (n = 3 per group, ∗p < 0.05, Student’s *t* test). (D) PCoA analysis using the Bray–Curtis dissimilarity index. (E) Spearman correlations between the *Ucp1* expression normalized to *Gtf2b* in IWAT and the relative abundance of bacterial families. Data of postnatal day 17 and day 22 were used.

Next, we examined the postnatal day-dependent change in gut microbiota composition by 16S rRNA gene-based metagenomic analysis. DNA was extracted from the caecum contents, and the sequence of the V3–V4 region amplified by PCR was examined by next-generation sequencing and then used to analyze the population of bacterial species. Notably, the amount of DNA extracted from the caecum of day 12 pups was low probably because of unmatured microbiota, and only two samples could be analyzed. Given the low sample number, the data of day 12 were shown as a reference and excluded in the following analyses. The relative taxonomic abundance of bacterial phyla is shown in Figure 2A. We observed the drastic changes in dominant bacteria at the phylum level among postnatal days: dominant bacteria included the Firmicutes phylum on day 12 (approximately 83%) but changed to the Bacteroidetes phylum on day 17 (79.1% ± 1.7%) (Figure 2B). After weaning, the relative abundance of the Bacteroidetes phylum was significantly reduced (28.7% ± 10.2%), and the relative abundance of the Firmicutes phylum significantly increased (41.9% ± 1.0%). Alpha diversity analysis, including Chao1 and Shannon indexes that indicate species richness and evenness, respectively, showed a significant increase on day 22 compared with day 17 (Figure 2C). Principal coordinate analysis (PCoA) using the Bray–Curtis index showed that the gut microbiota composition of postnatal days 12, 17, and 22 was clustered differently (Figure 2D). These results clearly indicate that the composition of gut microbiota differs among the three postnatal days. Next, we examined the relation between the bacteria at the family level and the *Ucp1* expression in IWAT on day 17 and day 22 and found that three bacterial families show significant correlation (Figure 2E): Porphyromonadaceae of the Bacteroidetes phylum (r = 0.74, p = 0.002) and Enterobacteriaceae of the Proteobacteria phylum (r = 0.67, p = 0.006) had a strong positive correlation with *Ucp1* level, whereas Ruminococcaceae of the Firmicutes phylum (r = −0.54, p = 0.038) had a negative correlation with *Ucp1* level. These results indicate the possible involvement of these bacterial families in the induction of beige adipocytes during the postnatal period.

### Effect of maternal antibiotic treatment on postnatal browning of white adipose tissue in pups

**Figure 3:**
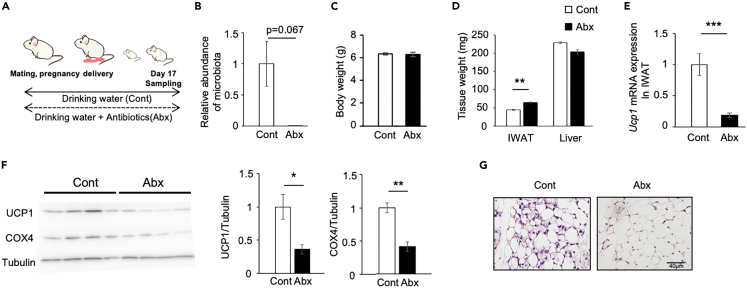
Effect of maternal antibiotic treatment on postnatal browning of white adipose tissue in pups (A) Experimental design and procedures. A/J female mice were given drinking water with (Abx) or without (Cont) antibiotics from mating to the sampling day. Pups were euthanized on postnatal day 17, and tissue samples were obtained. (B) Relative abundance of microbiota was analyzed by amplifying the V3–V4 region of 16S rRNA using real-time PCR. The expression values were normalized to *Gtf2b* and expressed relative to the value of the control group. Values represent means ± SEM (n = 4 per group, Student’s *t* test). (C and D) Effect of maternal antibiotic treatment on the weights of the body, inguinal white adipose tissue (IWAT), and liver. Values represent means ± SEM (n = 14 per group, ∗∗p < 0.01, Student’s *t* test). (E) Gene expression of *Ucp1* in IWAT. The expression values were normalized to *Actb* and expressed relative to the value of the control group. Values represent means ± SEM (n = 7–9 per group, ∗∗∗p < 0.001, Student’s *t* test). (F) Western blotting analysis of UCP1 and COX4 protein levels in IWAT. The expression values were normalized to tubulin and expressed relative to the value of the control group. Values represent means ± SEM (n = 4 per group, ∗p < 0.05, ∗∗p < 0.01, Student’s *t* test). (G) Histological images of IWAT. Scale bar: 40 μm.

In examining the role of gut microbiota directly, antibiotics were administered to dam to disrupt gut microbiota formation in pups (Figure 3A). Pups of the control dam (control group) and the antibiotic-treated dam (Abx group) were examined on day 17, when the expression level of *Ucp1* peaked (Figure 1E). The suppression of gut microbiota formation in pups was confirmed by the amplification of the V3–V4 region of 16S rRNA by real-time PCR (Figure 3B). BW (Figure 3C) and the weight of liver (Figure 3D) were similar in both groups; however, IWAT weight was significantly higher in the Abx group than in the control group. The level of *Ucp1* mRNA (Figure 3E) and the protein level of UCP1 and COX4 (Figure 3F) significantly decreased in the Abx group. In addition, histological analysis showed less multilocular adipocytes in the Abx group (Figure 3G), indicating the suppression of beige adipocyte induction. These results suggest that gut microbiota formation is necessary for the postnatal browning of IWAT.

### Effect of antibiotic treatment on bile acid composition in pups

**Figure 4:**
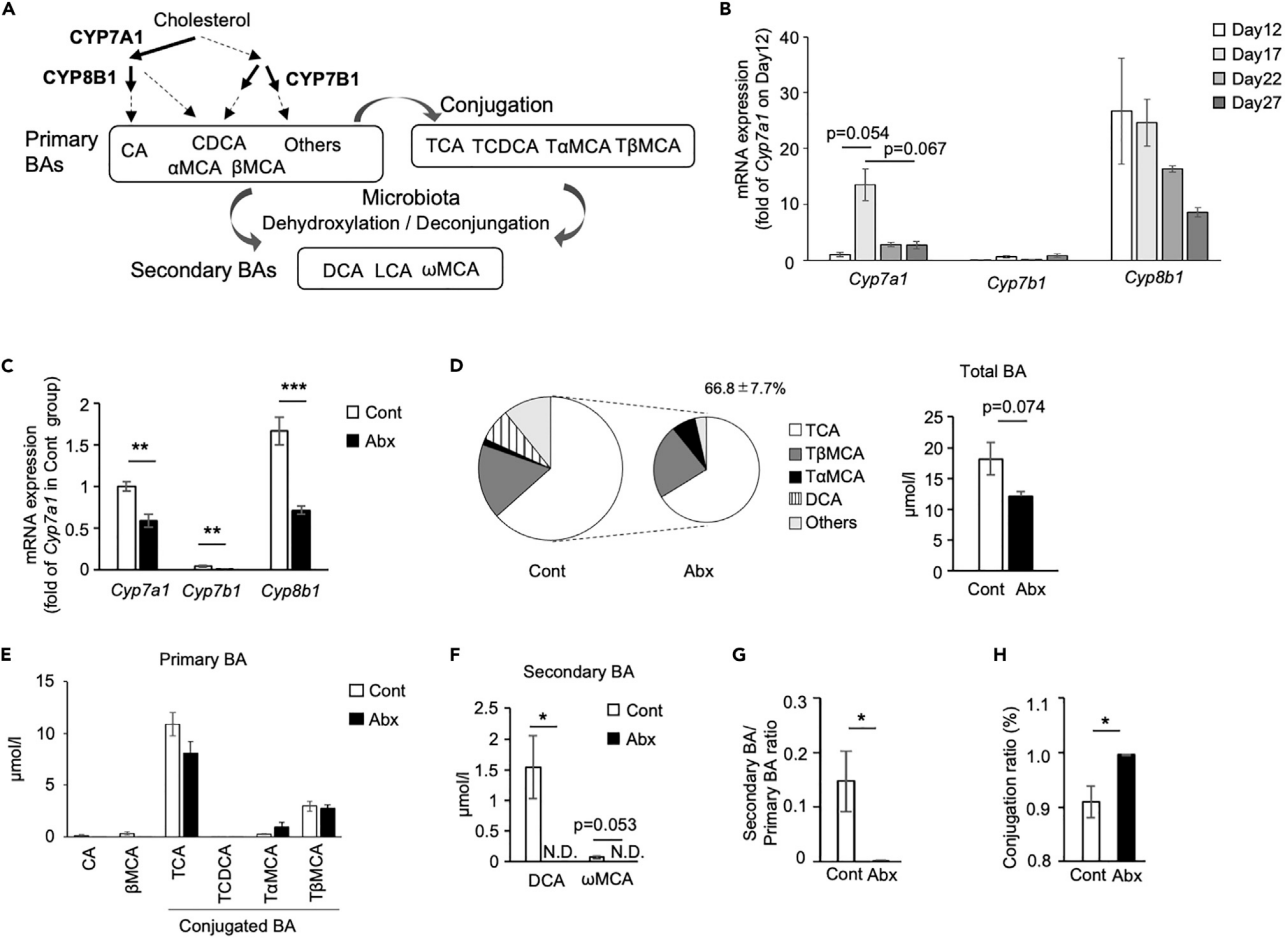
Effect of antibiotic treatment on bile acid composition in pups (A) Diagram of the bile acid (BA) metabolism pathway. (B) Postnatal changes in the expression of genes for key enzymes during BA synthesis in the liver. The expression values were normalized to *Actb* and expressed relative to the value of *Cyp7a1* on day 12. All values represent means ± SEM (n = 4–10 per group, one-way ANOVA followed by Tukey’s Honest Significant Difference test). (C–H) Gene expression of *Cyp7a1*, *Cyp7b1*, and *Cyp8b1* in the liver collected from pups in experiment described in Figure 3A. The expression values were normalized to *Actb* and expressed relative to the value of the control group. All values represent means ± SEM (n = 6–8 per group, ∗∗p < 0.01, ∗∗∗p < 0.001, Student’s *t* test). Serum BA composition and total BA concentration (D) as well as the concentration of primary (E) and secondary (F) BAs are shown. The ratio of secondary to primary BA (G) and conjugation (H) were calculated. All values represent means ± SEM (n = 6–8 per group, ∗p < 0.05, Student’s *t* test). CA, cholic acid; CDCA, chenodeoxycholic acid; MCA, muricholic acid; T, taurine-conjugated species.

Microbiota is closely related to bile acids (BAs) metabolism, and it was previously reported that oral administration of BAs increased the expression of *Ucp1* in IWAT in adult mice.^32^ In examining the involvement of BAs in the postnatal browning of IWAT, we analyzed the expression of enzymes related to BAs synthesis in the liver. As shown in Figure 4A, BAs are synthesized from cholesterol to form primary BAs and deconjugated by microbiota in the intestine to form secondary BAs. First, we examined the postnatal change in the gene expression level of the enzymes. The expression level of *Cyp7a1* transiently increased on day17 (Figure 4B), which was consistent with the timing of beige adipocyte induction in IWAT (Figure 1E). The *Cyp7b1* expression level was low, and it showed no specific changes from days 12–27. The *Cyp8b1* expression level was highest among the three genes and tended to decrease in a postnatal day-dependent manner. Next, we analyzed the liver of the Abx group and found that the expression levels of *Cyp7a1*, *Cyp7b1*, and *Cyp8b1* were all significantly low in the Abx group (Figure 4C). Subsequently, we checked the difference in BA profiles in serum using LC/MS. The total amount of BAs was reduced in the Abx group to 66.8% ± 7.7% of that in the control group, although the change was not statistically significant (p = 0.07, Figure 4D). There was no significant difference in primary BAs between the two groups, including taurocholic acid (TCA), which was a major conjugated BA in both groups (Figures 4D and 4E). Notably, secondary BA of deoxycholic acid (DCA), a major secondary BA in the control group, and ωMCA were not detected in the Abx group (Figure 4F). Consistently, the ratio of secondary BA to primary BA significantly decreased (Figure 4G), whereas the conjugation ratio significantly increased in the Abx group (Figure 4H). These results indicate that Abx treatment of dam reduced the synthesis of primary BA in liver and the secondary BA in intestine as well, resulting in the lower level of BAs in serum in pups. Collectively, it is suggested that the postnatally formed microbiota induce beige adipocytes through the induction of BA synthesis.

### Effect of maternal high-fat diet feeding on WAT browning in pups

**Figure 5:**
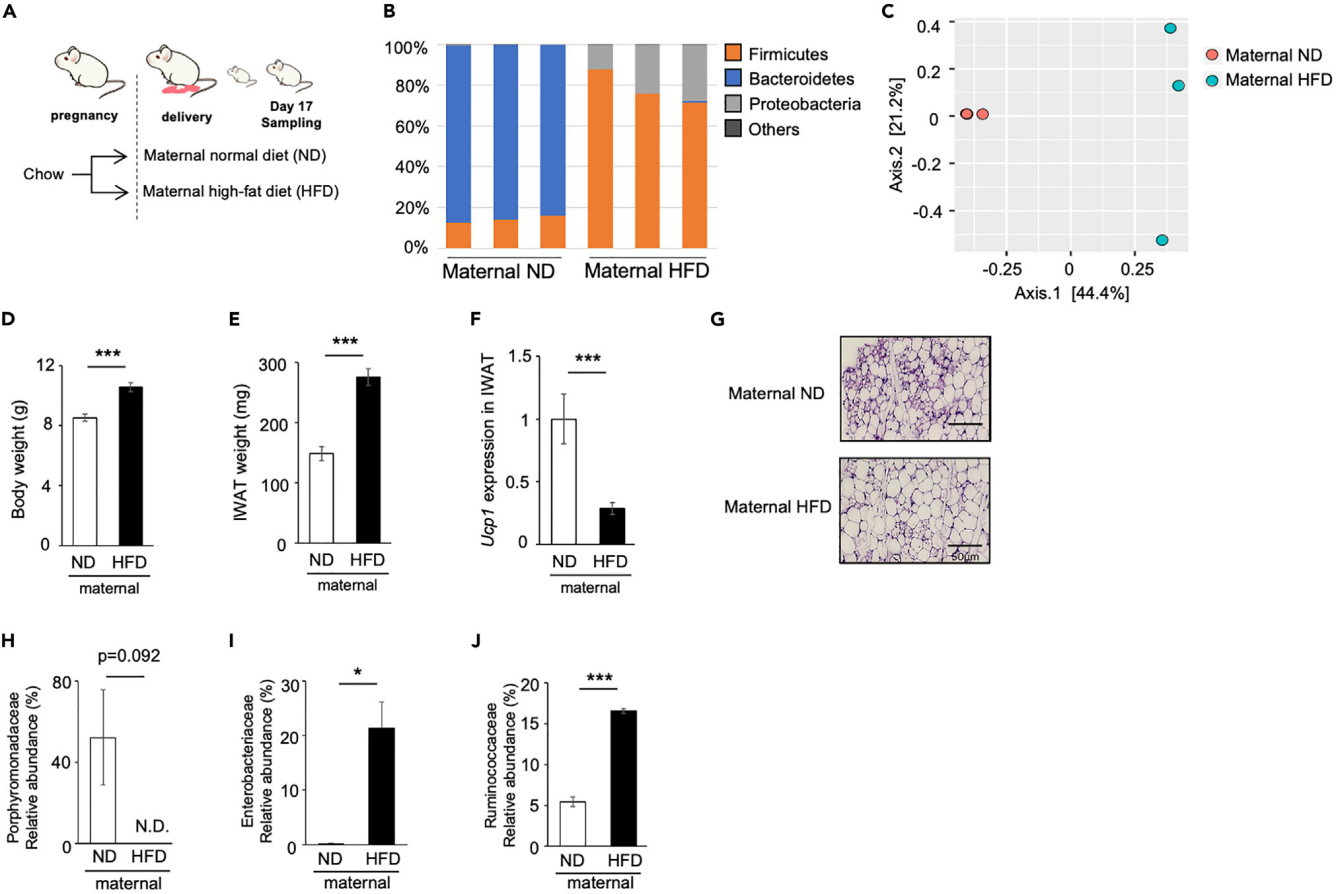
Effect of maternal high-fat diet feeding on WAT browning in pups (A) Experimental design and procedures. A/J female mice were given a normal (ND) or high-fat diet (HFD) from the day of delivery. Pups were euthanized on postnatal day 17, and tissue samples were obtained. (B) The relative taxonomic abundance of bacterial phyla in the caecum of pups. (C) PCoA analysis using the Bray–Curtis dissimilarity index. (D–F) Body weight (D) and inguinal white adipose tissue (IWAT) weight (E) of pups. Gene expression of *Ucp1* was analyzed (F). The expression values were normalized to *Actb* and expressed relative to the value of the ND group. Values represent means ± SEM (n = 18–20 per group, ∗∗∗p < 0.001, Student’s *t* test). (G) Histological images of IWAT. Scale bar: 50 μm. (H–J) Relative abundance of microbiota was analyzed by metagenomic analysis. Values represent means ± SEM (n = 3 per group, ∗p < 0.05, ∗∗∗p < 0.001, Student’s *t* test).

To eliminate the direct effect of antibiotics on WAT browning^33^^,^^34^ we examined the role of microbiota in postnatal browning of WAT in different conditions. Because the postnatal formation of microbiota is known to be affected by the breast milk composition,^35^^,^^36^ dams after the delivery were fed with normal diet (ND) or high-fat diet (HFD) and WAT browning in pups was examined (Figure 5A). Consequently, maternal HFD feeding drastically changed microbiota composition in pups (Figure 5B): the dominant bacteria at the phylum level were Firmicutes in maternal HFD group (78.3% ± 4.8%), whereas the Bacteroidetes phylum in the maternal normal diet (ND) group (85.5% ± 1.0%). PCoA also showed that gut microbiota composition of the maternal ND and HFD groups were clustered differently (Figure 5C). Next, we examined the effect of maternal ND/HFD feeding on postnatal WAT browning. On day 17, BW and IWAT weight of pups were larger in the maternal HFD group than those in the maternal ND group (Figures 5D and 5E). The expression of *Ucp1* was significantly low (Figure 5F) and a decrease in multilocular adipocytes was observed in the maternal HFD group (Figure 5G), indicating the suppression of postnatal WAT browning by maternal HFD. We examined the relative abundance of three bacterial family that showed significant correlation with *Ucp1* expression in Figure 2E. The abundance of Porphyromonadaceae and Enterobacteriaceae, both of which have shown positive correlation with *Ucp1* expression (Figure 2E), decreased and increased, respectively, in the maternal HFD group compared to the maternal ND group (Figures 5H and 5I). The abundance of Ruminococcaceae, which have shown negative correlation with *Ucp1* expression (Figure 2E), significantly increased by maternal HFD feeding (Figure 5J).

### Effect of maternal high-fat diet feeding on bile acid composition in pups

**Figure 6:**
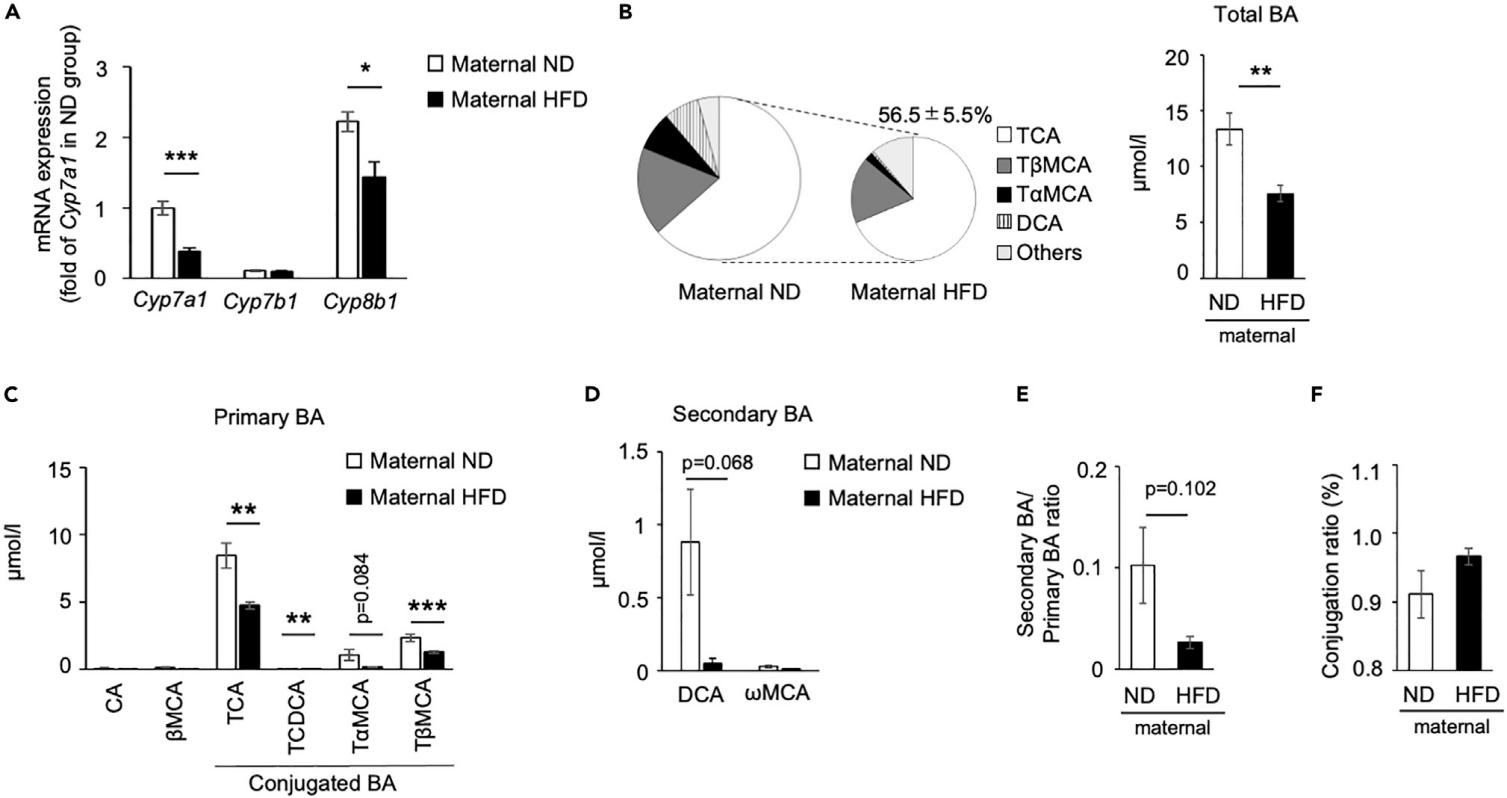
Effect of maternal high-fat diet feeding on bile acid composition in pups (A) Gene expression of *Cyp7a1*, *Cyp7b1*, and *Cyp8b1* in the liver collected from 17-day-old pups from dam fed with normal diet (ND) or high-fat diet (HFD) after delivery. The expression values were normalized to *Gtf2b* and expressed relative to the value of *Cyp7a1* in the ND group. All values represent means ± SEM (n = 5–6 per group, ∗p < 0.05, ∗∗∗p < 0.001, Student’s *t* test). BA composition and total BA (B) as well as the concentration of primary (C) and secondary (D) BAs in serum were measured. The ratio of secondary to primary BAs (E) and conjugation (F) were calculated. All values represent means ± SEM (n = 6 per group, ∗∗p < 0.01, ∗∗∗p < 0.001, Student’s *t* test). CA, cholic acid; CDCA, chenodeoxycholic acid; MCA, muricholic acid; T, taurine-conjugated species.

Maternal HFD feeding decreased the expression of *Cyp7a1* and *Cyp8b1*, but not *Cyp7b1*, in the liver (Figure 6A). BA composition was also affected by maternal HFD feeding, and total BA concentration was decreased to 56.5% ± 5.5% of the maternal ND group (Figure 6B). In primary BAs, no significant difference was observed in CA and βMCA, however, maternal HFD feeding significantly decreased the conjugated BAs, including the most and second major BAs, TCA, and TβMCA (Figure 6C). Similar to the Abx treatment (Figure 4), maternal HFD feeding decreased secondary BAs (DCA and ωMCA; Figure 6D). The ratio of secondary BA to primary BA and conjugation were not changed (Figures 6E and 6F). Collectively, maternal HFD feeding suppressed postnatal WAT browning with changes in the composition of microbiota and BAs.

### Effect of microbiota transplantation on postnatal WAT browning in pups

**Figure 7:**
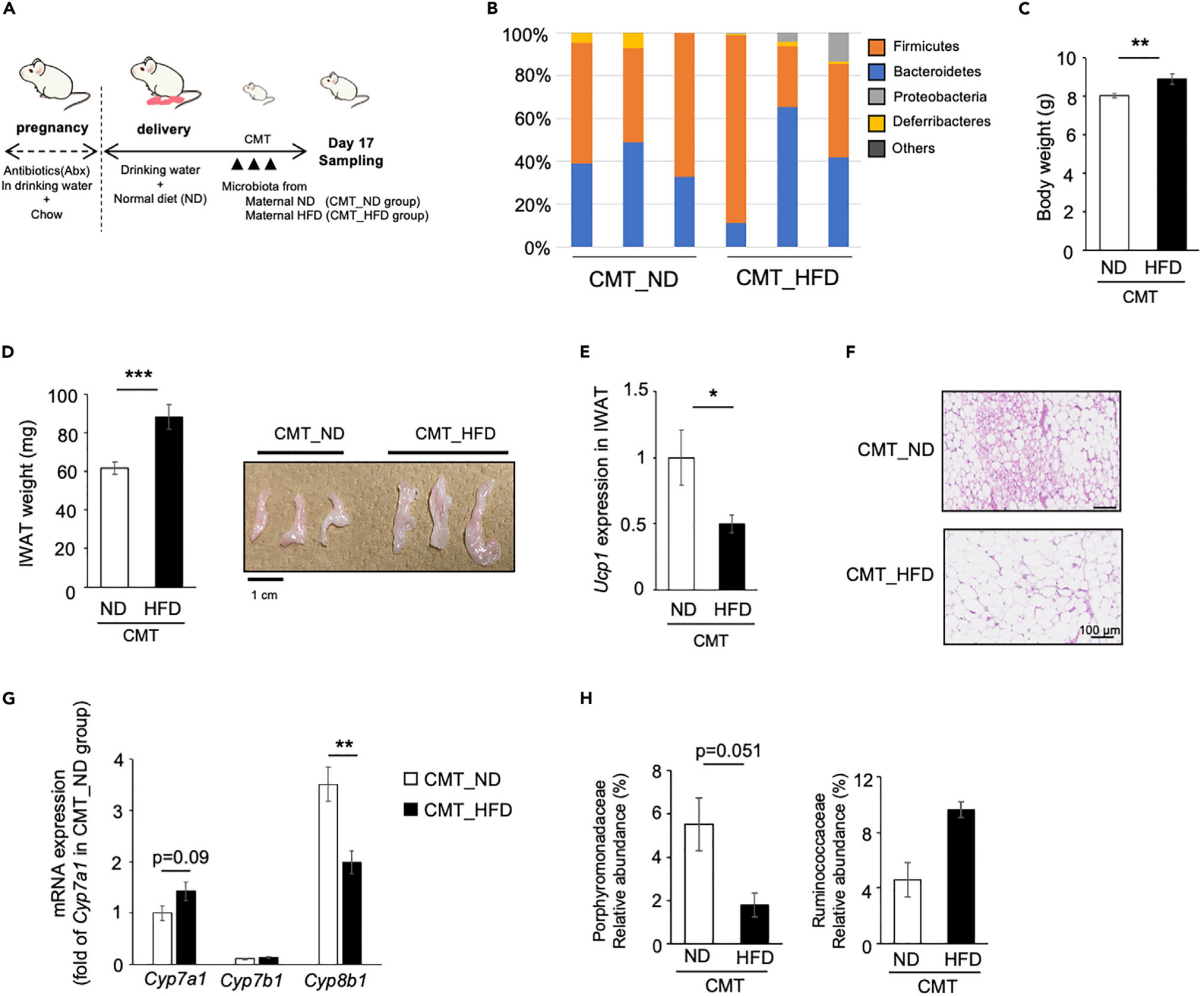
Effect of microbiota transplantation on postnatal WAT browning in pups (A) Experimental design and procedures. A/J female mice were given drinking water with antibiotics from mating to the day of delivery. After delivery, dams were fed with normal diet. Pups were transplanted on postnatal days 5, 10, and 15 with microbiota obtained from the caecum of 17-day-old pups in maternal ND or HFD feeding experiments described in Figure 5 (CMT-ND CMT-HFD groups, respectively). Pups were euthanized on postnatal day 17, and tissue samples were obtained. (B) The relative taxonomic abundance of bacterial phyla in the caecum of pups. (C–E) Body weight (C) and inguinal white adipose tissue (IWAT) weight (D) of pups. Gross appearance of IWAT is shown. Scale bar: 1 cm. Values represent means ± SEM (n = 12–14 per group, ∗∗p < 0.01, ∗∗∗p < 0.001, Student’s *t* test). Gene expression of *Ucp1* was analyzed (E). The expression values were normalized to *Gtf2b* and expressed relative to the value of the CMT-ND group. Values represent means ± SEM (n = 6 per group, ∗p < 0.05, Student’s *t* test). (F) Histological images of IWAT. Scale bar: 100 μm. (G) Gene expression of *Cyp7a1*, *Cyp7b1*, and *Cyp8b1* in the liver was measured. The expression values were normalized to *Gtf2b* and expressed relative to the value of *Cyp7a1* in the CMT-ND group. All values represent means ± SEM (n = 6 per group, ∗∗p < 0.01, Student’s *t* test). (H) Relative abundance of microbiota was analyzed by metagenomic analysis. Values represent means ± SEM (n = 3 per group, Student’s *t* test).

In confirming the role of microbiota in postnatal WAT browning, cecal microbiota transplantation (CMT) experiment was conducted. Pups of the Abx-treated dams were transplanted with the cecal microbiota of the 17-day-old pups in the maternal ND or HFD groups (CMT_ND and CMT_HFD group, respectively; Figure 7A). The microbiota composition on day 17 was not much different between the CMT_ND and CMT_HFD group (Figure 7B), and they were different from those in donor maternal ND or HFD mice (Figure 5B). Of interest, transplantation of the microbiota from maternal HFD mice resulted in higher BW and IWAT weight (Figures 7C and 7D). In addition, *Ucp1* expression was reduced (Figure 7E), and smaller number of beige adipocytes were observed (Figure 7F) in the CMT_HFD group compared to the CMT_ND group. *Cyp8b1* expression in the liver significantly decreased in the CMT_HFD group, although *Cyp7a1* failed to show significant difference between the two groups (Figure 7G). The abundance of Porphyromonadaceae tended to decrease in the CMT_HFD group (p = 0.051), but no difference in the abundance of Ruminococcaceae was observed between the two groups (Figure 7H).

## Discussion

In this study, we investigated the mechanism underlying the postnatal induction of beige adipocytes during the early postnatal period in mice. We found that UCP1-expressing beige adipocytes were induced in WAT, peaking at postnatal day 17, but they disappeared after weaning. Considering that microbiota composition drastically changes after birth and weaning, we hypothesized that postnatal changes in microbiota composition play a role in postnatal WAT browning. The disruption of postnatal microbiota formation caused by the use of antibiotics suppressed the induction of beige adipocytes in pups. Maternal HFD feeding resulted in the compositional change of microbiota in pups and suppressed WAT browning. This suppression was also observed after the transplantation of microbiota from pups of the maternal HFD group. These results indicate that microbiota formation and composition before weaning are important for postnatal WAT browning.

The gut microbiota is postnatally shaped and stabilized under the influence of breast milk and environment during pre-weaning, and it further changes after weaning depending on the diet and other various physiological conditions.^37^ In this study, the microbiota of pups was drastically changed from day 12 to day 17: Firmicutes, a major microbiota on day 12 accounting for approximately 80% of all microbiota, decreased to below 20% on day 17, with substitutional increase of Bacteroidetes being around 80%. Weaning also changed microbiota composition, including Bacteroidetes (29%), Firmicutes (42%), and Deferribacteres (27%). Gut microbiota has been reported to have a significant effect on host metabolism.^38^ Firmicutes phylum and Bacteroidetes phylum are two major phyla in the composition of gut microbiota, and multiple studies have suggested the ratio of the two phyla is associated with obesity in mice and humans.^39^ Although there is still controversial in humans,^40^ the abundance of Bacteroidetes phylum decreased in mice fed a HFD,^41^^,^^42^ whereas the increase in the abundance of Bacteroidetes phylum inhibited body weight gain in diet-induced obese mice.^43^ In this study, the abundance of the Bacteroidetes phylum showed a clear correlation with WAT browning: it increased by postnatal day 17 when *Ucp1* mRNA expression reached maximum and decreased after weaning with low *Ucp1* expression. This correlation is consistent with a previous report, that is, cold exposure increased the abundance of the Bacteroidetes phylum in the caecum, followed by the induction of IWAT browning.^28^ In this study, Pearson’s correlation test revealed the positive correlation between the expression of *Ucp1* and Porphyromonadaceae, which belongs to the Bacteroidetes phylum. This bacterial species was not detected in pups of the maternal HFD group, which showed suppressed WAT browning, and the transplantation of their microbiota suppressed WAT browning, indicating the important role of Porphyromonadaceae in the induction of beige adipocytes. Previous reports also suggested the positive relation between Porphyromonadaceae abundance and *Ucp1* expression in epididymal WAT in HFD-induced obese mice after short-term fasting.^44^ The association between the abundance of Porphyromonadaceae and weight loss is also reported in human.^45^ Thus, it is plausible that the Porphyromonadaceae family can improve obesity through the induction of brown and beige adipocytes. Although the mechanism by which Porphyromonadaceae induces *Ucp1* expression in WAT is unknown, its ability to produce butyric acid might be involved.^44^ The supplementation of butyric acid in diet-induced obese mice have been reported to improve insulin resistance and induce UCP1 expression in BAT through the activation of PGC-1a.^46^ In addition, cold stimulation induced the increase of butyrate-producing bacteria.^47^ However, in this study, the butyrate level in feces of pups failed to show any difference between the maternal ND and HFD groups (Figure S1A). Thus, it is likely that Porphyromonadaceae induces WAT browning by mechanisms other than its production of butyrate.

Apart from Porphyromonadaceae, we found a negative correlation between Ruminococcaceae, which belongs to the Firmicutes phylum, and the expression of *Ucp1* during peri-weaning. The abundance of Ruminococcaceae was significantly increased in the maternal HFD group with the significant reduction of *Ucp1* expression; however, it was not correlated with *Ucp1* expression in CMT experiment. Previous reports have shown the increase in the abundance of Ruminococcaceae after cold exposure^48^ or the positive correlation with thermogenic activity of BAT.^49^ Collectively, it is unlikely that the Ruminococcaceae play a suppressive role in postnatal Ucp1 induction in WAT. Another bacterial family showed a positive correlation with the expression of *Ucp1*. Enterobacteriaceae, which belongs to the Proteobacteria phylum, is dominant in early neonatal gut microbiota in human^37^ and mice.^50^ In this study, the abundance of Enterobacteriaceae was not correlated with *Ucp1* expression in the maternal HFD group, and Enterobacteriaceae was not detected after CMT despite Ucp1 induction. Enterobacteriaceae seems to be dispensable for postnatal WAT browning.

One of the bacteria that has been reported to be associated with WAT browning is *Lactobacillus*. The administration of probiotic bacterium belonging to *Lactobacillus* to obese mice enhanced thermogenic gene expression, including *Ucp1* in IWAT, and increased body temperature.^51^ Another recent study suggested that treatment of *Lactobacillus* prevented weight gain in obese mice and upregulated *Ucp1* expression in WAT.^52^ However, our result did not show any significant correlation between Lactobacillaceae abundance and *Ucp1* expression in IWAT. The discrepancy might be because of the age-dependent difference between preweaning and adult mice, or it would be difficult to explain by one species because intestinal bacteria often act cooperatively with one another. Thus, further study is necessary to identify which bacteria are responsible for the induction of beige adipocytes in IWAT during the neonatal period. Collectively, this study revealed the indispensable role of microbiota formation in the postnatal browning of WAT in mice.

As a factor involved in the mechanism by which microbiota induces browning, we focused on BAs. BAs are synthesized in the liver from cholesterol through two pathways, classical and alternative, by enzymes such as cytochrome P450 (CYP) hydroxylase to form primary BAs. Conjugated with taurine or glycine in the liver, primary BAs were deconjugated by intestinal bacteria to form secondary BAs.^53^ The role of BAs in *Ucp1* induction is supported by previous reports, that is, the administration of BAs induced thermogenetic gene expression in IWAT,^32^ and the activation of Transmembrane G protein-coupled receptor 5 (TGR5), a BA receptor, promoted the recruitment of beige adipocytes in WAT in adult mice.^54^ The oral supplementation of CDCA increased human BAT activity and whole-body energy expenditure.^55^ Consistent with a previous report,^56^ the mRNA level of CYP7A1, the first and rate-limiting enzyme in the classical pathway, increased transiently on day 17, showing a similar postnatal-day-dependent expression pattern to *Ucp1* in IWAT. The low mRNA level of CYP7B1 indicated the minor role of the alternative pathway in this period. The expression of *Cyp7a1* and *Cyp8b1* in the classical pathway significantly decreased by maternal Abx treatment and maternal HFD feeding, resulting in the low blood concentration of total BA. Considering that TGR5 is preferentially activated by secondary BAs, the low level of DCA in both groups likely contributes to the suppression of WAT browning. However, changes in microbiota might also lead to the modification of BA synthesis system and the suppression of browning in parallel, without any causal relationship between the two phenomena. Further study is required to confirm the role of BAs in postnatal WAT browning, such as an experiment using TGR5-knockout mice.

In this study, we found that microbiota formation after birth is important to postnatal WAT browning. In addition, weaning-related changes in microbiota composition may be related to the disappearance of beige adipocytes. Yu et al. reported that breast milk intake induces beige adipocytes through breast milk-specific lipid species, alkylglycerols, which is metabolized by adipose tissue macrophages to platelet-activating factors that activate IL-6/STAT3 signaling to induce *Ucp1* expression in adipocytes.^57^ Considering that maternal HFD feeding largely affects fatty acid composition in breast milk,^58^^,^^59^^,^^60^ maternal HFD feeding may change the content of lipids such as alkylglycerols in breast milk, thereby affecting WAT browning. However, in this study, the WAT browning phenotype of the maternal HFD group was inherited by the transplantation of gut microbiota. Thus, the microbiota-mediated mechanism, rather than the direct action of breast milk-derived factor on WAT, is involved in the suppression of WAT browning in the maternal HFD group. Considering that alkylglycerols have shown *Lactobacillus* proliferation *in vitro,*^61^ the indirect effect of alkylglycerols or other lipid species through their effect on microbiota can be assumed, although we did not detect a correlation between *Lactobacillus* and beige adipocytes. On the contrary, in this study, maternal HFD feeding after delivery failed to change microbiota composition in dams (Figure S1B), although it changed that in pups. Therefore, microbiota changes in pups of the maternal HFD group was not because of the transmission from their mother. The dams were fed an HFD only after delivery; thus, the quality of the diet consumed by lactating mothers may directly contribute to the formation of the gut microbiota via breast milk, thereby affecting WAT browning. Collectively, our study provides new insights into the diet of lactating mothers, which influences the adipose tissue remodeling of their offspring through the modulation of microbiota.

The physiological significance of postnatal WAT browning remains unclear. In rodents, WAT starts development before birth, but it is less advanced at birth.^62^ This finding is contrary to BAT, which develops in fetus and already functionally matured at birth^63^ with a further enhancement of its thermogenic function after birth.^64^^,^^65^ The browning experience of WAT during pre-weaning may affect WAT function after growth. The ablation of beige adipocytes formed during peri-weaning resulted in the reduction of cold-induced browning in adult mice.^66^ Thus, the adequate induction of beige adipocytes during the postnatal period may alter the metabolic phenotype of WAT, and the effect persists into later life. Increasing evidence on human and animal studies indicates that maternal obesity during gestation and lactation predisposes offspring to obesity and metabolic diseases in later life.^67^^,^^68^ A limiting number of studies have investigated the effect of HFD only during lactation, but animal studies have shown that HFD feeding to lactating dams predisposes the offspring for obesity and related metabolic abnormalities by impairing neurodevelopment,^69^ leptin sensitivity,^70^ or inflammation.^71^ Considering that beige adipocytes have been revealed not only to reduce obesity but also to improve glucose and lipid metabolism, if the phenotype of WAT regarding browning capacity in pre-weaning is sustained over to later life, then it will have a major impact on the metabolism of individuals. In addition, the number of brown/beige adipocytes varies greatly with gender and age in mice and humans.^72^ Therefore, understanding the mechanism that defines these individual differences is necessary for the establishment of effective obesity countermeasures. Future research must also investigate the significance of postnatal WAT and its role on adipose tissue development and post-growth metabolism.

In this study, we show that WAT undergoes browning during the preweaning period in association with the formation of microbiota in mice. Considering that the induction mechanism of postnatal browning has been suggested to be independent of sympathetic nerve activity,^20^^,^^21^^,^^22^ unlike in adults, our findings may contribute to the elucidation of a sympathetic stimulation-independent beige adipocyte induction mechanism.

### Limitations of the study

It is possible that the gut bacteria related to the induction and the disappearance of beige adipocytes are different. Since we used data of day 17 and day 22 for the correlation analysis with Ucp1 because of the low amount of DNA extracted from the caecum of day 12 pups, it may lead to the extraction of the gut bacteria associated only with the disappearance of beige adipocytes.

We revealed that change in the postnatal formation of gut microbiota caused the modification of BA synthesis system and the suppression of WAT browning, yet the causal relationship between the two remains unclear. To confirm the role of bile acids in postnatal WAT browning, further research, such as an experiment using TGR5-knockout mice, is required.

## STAR★Methods

### Key resources table

**Table:** 

REAGENT or RESOURCE	SOURCE	IDENTIFIER
**Antibodies**		

Rabbit polyclonal anti-UCP1	Abcam	Cat# ab10983; RRID:AB_2241462
Mouse monoclonal anti- OxPhos Complex IV subunit IV	Thermo Fisher Scientific	Cat# A21348; RRID:AB_2535839
Mouse monoclonal anti-α-Tubulin	Sigma-Aldrich	Cat# T9026; RRID:AB_477593
Polyclonal goat anti-rabbit IgG HRP-linked antibody	Cell Signaling Technology	Cat# 7074; RRID:AB_2099233
Polyclonal horse anti-mouse IgG HRP-linked antibody	Cell Signaling Technology	Cat# 7076; RRID:AB_330924

**Biological samples**		

Mouse adipose tissue	This paper	NA
Mouse serum	This paper	NA
Mouse cecal content	This paper	NA
Mouse liver	This paper	NA

**Chemicals, peptides, and recombinant proteins**		

Neomycin	FUJIFILM Wako Pure Chemical Corporation	Cat# 146-08871
Vancomycin	FUJIFILM Wako Pure Chemical Corporation	Cat# 226-0130
Metronidazole	FUJIFILM Wako Pure Chemical Corporation	Cat# 132-18061
Bacitracin	FUJIFILM Wako Pure Chemical Corporation	Cat# B106500
Ceftazidime	FUJIFILM Wako Pure Chemical Corporation	Cat# C1635
Gentamycin	FUJIFILM Wako Pure Chemical Corporation	Cat# 079-02973
Streptomycin	Meiji Seika Pharma	Cat#4987222665643
Penicillin	Meiji Seika Pharma	Cat#4987222637756
Ciprofloxacin	Tokyo Kasei	Cat# C2227

**Critical commercial assays**		

QIAamp Fast DNA Stool Mini Kit	Qiagen	Cat# 51504

Deposited data		

Raw data	https://www.ncbi.nlm.nih.gov/	PRJNA985901

**Experimental models: Organisms/strains**		

Mouse A/J	Japan SLC Inc	NA

**Oligonucleotides**		

Primers for this study, see table in "Real-time PCR" section	This paper	NA

**Software and algorithms**		

QIIME2	Bolyen et al., 2019	Version 2021.8

### Resource availability

#### Lead contact

Further information and requests for resources and reagents should be directed to and will be fulfilled by the lead contact, Yuko Okamatsu-Ogura (y-okamatsu@vetmed.hokudai.ac.jp)

#### Materials availability

This study did not generate new unique reagents.

### Experimental model and study participant details

The experimental procedures and care of animals were approved by the Animal Care and Use Committee of Hokkaido University (Sapporo, Japan). All experiments using mice were conducted in an animal facility approved by the Association for Assessment and Accreditation of Laboratory Animal Care International. A/J mice were purchased from Japan SLC Inc (Hamamatsu, Japan) and housed in plastic cages placed in a temperature-controlled room at 22°C ± 4°C with a 12:12 h light/dark cycle and given free access to laboratory chow (CE-2, Oriental Yeast, Tokyo, Japan) and tap water. After 1-week acclimation period, male and female mice were mated by putting three female (6 weeks old) into a cage with a male (7 weeks old). Once mating was confirmed by plug check, male was removed. On the next day of the delivery, litter size was standardized to 4–5 pups per litter. Pups were euthanized by intraperitoneal injection of Ional sodium (secobarbital sodium; Nichi-Iko Pharmaceutical Co., Toyama, Japan) at the indicated day and blood was collected after decapitation. Inguinal white adipose tissue (IWAT), stomach, caecum, and liver were collected.

For antibiotic (Abx) treatment, female mice were given a drinking water with or without antibiotics [100 μg/mL Neomycin, 50 μg/mL Vancomycin, 100 μg/mL Metronidazole, 1 mg/mL Bacitracin, 100 μg/mL Ceftazidime, and 170 μg/mL Gentamycin purchased from FUJIFILM Wako Pure Chemical Corporation (Osaka, Japan), 50 μg/mL Streptomycin and 100 U/ml Penicillin purchased from Meiji Seika Pharma (Tokyo, Japan), and 125 μg/mL Ciprofloxacin (Tokyo Kasei, Tokyo, Japan)^73^ from the day of the start of mating throughout of the experiment. For maternal high-fat diet (HFD)-feeding experiment, dams were given a normal diet (ND) (10 kcal % fat, D12450B, Research Diet, New Brunswick, NJ, USA) or HFD (40 kcal% fat, D12451, Research Diets) from the day of delivery. For cecal microbiota transplantation (CMT) experiment, female mice were treated with antibiotics from the day of the start of mating until the delivery. After the delivery, dams were given a drinking water without antibiotics, and fed with ND. Pups were orally administrated with microbiota solution (60 μL/pup) on postnatal day 5, 10, and 15. Microbiota solution was prepared from caecum contents of 17-day-old pups from the maternal ND or HFD groups as follows. The caecum contents were suspended in CMT buffer (37.47 mM KH_2_PO_3_, 34.45 mM K_2_HPO_4_, 113.87 mM L-cysteine monohydrochloride, 351.15 mM Tween 80) at 10 times their wet weight, and centrifuged (4°C, 30 × g, 2 min). The supernatant was mixed with one-tenth volume of glycerol and stored at −80°C until use.

### Method details

#### Histological analysis

Tissue specimens were fixed in 10%-buffered formalin and embedded in paraffin according to the conventional method, cut into 3-μm-thick sections, and stained with hematoxylin and eosin.

#### Real-time PCR

Total RNA was extracted from tissues stored in RNAlater (Thermo Fisher Scientific, Gaithersburg, MD, USA) using TRIzol reagent (Thermo Fisher Scientific) according to the manufacturer’s instructions, and reverse-transcribed using a 15-mer oligo (dT) adaptor primer and M-MLV reverse transcriptase (Promega, Madison WI, USA). Real-time PCR was conducted using a fluorescence thermal cycler (LightCycler system, Roche Diagnostics, Mannheim, Germany) and Brilliant III Ultra-Fast SYBR Green QPCR Mater Mixes (Agilent Technologies, Santa Clara, CA, USA). Absolute expression levels were determined using a standard curve using respective cDNA fragments as standards. The mRNA levels are expressed as relative values compared with *Actb* or *Gtf2b* mRNA levels. The primers used are listed in below table.

**Table 1:** Primer sequences for quantitative PCR and metagenomic analysis

*Actb*	Forward	5′-AAGTGTGACGTTGACATCCG -3′
	Reverse	5′- GATCCACACAGAGTACTTGC-3′
*Gtf2b*	Forward	5′-TGGAGATTTGTCCACCATGA-3′
	Reverse	5′-GAATTGCCAAACTCATCAAAACT-3′
*Ucp1*	Forward	5′-GGCCTCTACGACTCAGTCCA-3′
	Reverse	5′- TAAGCCGGCTGAGATCTTGT-3′
*Cyp7a1*	Forward	5′-ATTCCTGCAACCTTCTGGAG-3′
	Reverse	5′-TTGGCCAGCACTCTGTAATG-3′
*Cyp7b1*	Forward	5′-TCTCTTTGCCGCCACCTTAC-3′
	Reverse	5′-TTTCAGGGCCATGCCAAGAT-3′
*Cyp8b1*	Forward	5′-ATGATCGGTTCCTCAACCCG-3′
	Reverse	5′-GGCATGCTGTAGTGGTGGAT-3′
16S rRNA	341F	5′-TCGTCGGCAGCGTCAGATGTGTAT AGACAGCCTACGGGNGGCWGCAG-3′
	805R	5′-GTCTCGTGGGCTCGGAGATGTGTATAA GAGACAGGACTACHVGGGTATCTAATCC-3′

#### Western blotting

Tissues were homogenized in ice-cold RIPA buffer (50mM Tris-HCl pH 7.6, 0.15M NaCl, and 1% NP-40) containing cocktails of phosphatase inhibitor (Nacalai Tesque, Kyoto, Japan) and protease inhibitor (Sigma-Aldrich, St Louis, MO, USA). After centrifugation at 800 × g for 20 min at 4°C, the middle layer was taken as total protein to avoid taking the upper fat layer and used for western blotting analysis. In brief, the protein was separated by SDS-PAGE and transferred to a polyvinylidine fluoride membrane (Immobilon; Millipore, Bedford, MA, USA). After blocking with 5% skimmed milk (Morinaga Milk Industry Co., Tokyo, Japan), the membrane was incubated with a primary antibody overnight at 4°C. Primary antibodies against UCP1 (Abcam, Cambridge, UK), COX4 (Thermo Fisher Scientific), and Tubulin (Sigma, St. Louis, USA) were used. The bound antibody was visualized using a horseradish peroxidase-linked secondary antibody and an enhanced chemiluminescence system (GE Healthcare UK Ltd., Little Chalfont, Bucks, UK).

#### Metagenomic analysis

DNA was extracted from the cecal contents or feces using QIAamp Fast DNA Stool Mini Kit (Qiagen, Venlo, Netherland) according to the manufacturer’s instructions. V3-V4 region of 16S rRNA was amplified by PCR with the primers 341F and 805R listed in table in "Real-time PCR" section and KOD FX (TOYOBO, Osaka, Japan). Amplified PCR product was purified using Agencourt AMPure XP Beads (Beckman Coulter, CA, USA) and sent to Macrogen Japan Corp. (Tokyo, Japan) for next generation sequence analysis. The sequence data were analyzed using QIIME2^74^ and the taxonomy was assigned to each operational taxonomic unit (OTU) using the Greengenes reference database.^75^ PCoA was performed based on the Bray-Curtis index.

#### Bile acids (BAs) analysis

Plasma BA extraction and analysis were conducted using a Dionex UltiMate 3000 UPLC system (Thermo Fisher Scientific) according to our previous report.^76^^,^^77^ Briefly, 100μL of plasma samples was freeze-dried for later use. 25 nmol of 23-nor-5β-cholanic acid-3α,12α-diol (nordeoxycholic acid, NDCA) dissolved in ethanol was added as an internal standard. The samples with ethanol were homogenized and subjected to sonication and heating. After the centrifuge, the supernatant was collected and evaporated. The dried extracts were purified with an HLB cartridge (Waters, Milford, MA, USA) and reconstituted with methanol for analysis. Mass spectrometry was performed using an Orbitrap mass spectrometer Q Exactive^TM^ (Thermo Fisher Scientific) equipped with an electrospray ionization probe in the negative-ion mode. BA concentration was measured using NDCA as the internal standard.

#### Short chain fatty acid (SCFA) analysis

100 mg of cecal contents were collected and sent to Techno Suruga Laboratory (Shizuoka, Japan). Butyrate concentration was examined by Post-column pH Buffered Electric Conductivity Detection with liquid chromatography.

### Quantification and statistical analysis

Values are expressed as mean ± SEM. Statistical analyses were performed using Student’s *t* test or one-way analysis of variance (ANOVA) followed by the Tukey’s Honest Significance Difference post hoc test. The correlation between gut microbiota and mRNA levels of *Ucp1* was evaluated using Pearson correlation coefficient test. Statistical significance was set at p < 0.05.

## Data Availability

• Raw data generated from next-generation sequencing platform have been deposited at SRA and are publicly available as of the date of publication. Accession numbers are listed in the key resources table. All data reported in this paper will be shared by the lead contact upon request. • This paper does not report original code. • Any additional information required to reanalyze the data reported in this paper is available from the lead contact upon request.
