# Supplementary material for: White adipose tissue undergoes browning during preweaning period in association with microbiota formation in mice
Source: iScience. 2023 Jun 28;26(7):107239. doi: 10.1016/j.isci.2023.107239 (PMC10362363; doi:10.1016/j.isci.2023.107239)
Supplement: Document S1. Figure S1 [file mmc1.pdf]

iScience, Volume *26*

Supplemental information

White adipose tissue undergoes browning during preweaning period in association with microbiota formation in mice

Anju Tsukada, Yuko Okamatsu-Ogura, Emi Futagawa, Yuki Habu, Natsumi Takahashi, Mira Kato-Suzuki, Yuko Kato, Satoshi Ishizuka, Kei Sonoyama, and Kazuhiro Kimura

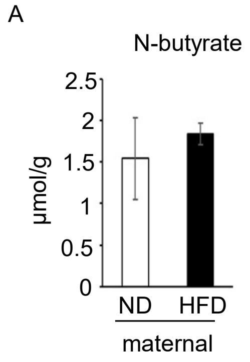

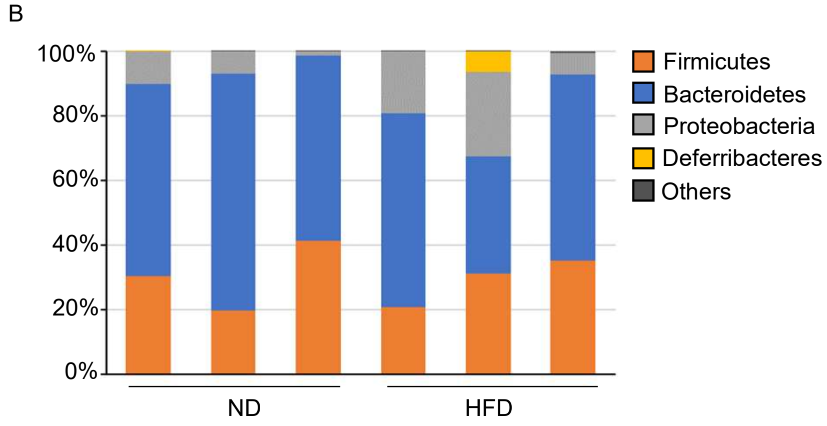

Figure S1. Effect of maternal high-fat diet feeding on WAT browning in pups, related to Figure 5

(A) The concentration of n-butyrate in feces of pups was measured. Values represent means ± SEM (n = 3 per group)
(B) The relative taxonomic abundance of bacterial phyla in the feces of dams.
